# Two perils of binary categorization: why the study of concepts can't afford true/false testing

**DOI:** 10.3389/fpsyg.2015.00168

**Published:** 2015-02-18

**Authors:** Greg Jensen, Drew Altschul

**Affiliations:** ^1^Department of Psychology, Columbia UniversityNew York, NY, USA; ^2^Department of Psychology, University of EdinburghEdinburgh, UK

**Keywords:** concepts, categorization, machine learning, animal cognition, comparative cognition

## 1. Introduction

Many claims about concept learning in animals rely on binary categorization tasks (Herrnstein et al., 1976; Freedman et al., 2001; Marsh and MacDonald, 2008). When subjects exceed chance levels of performance, they are alleged to have learned “the concept.” Critics are quick to point out that although subjects have learned *something*, confounds may explain performance more simply (Katz et al., 2007; Wright and Lickteig, 2010; Zentall et al., 2014). Despite a growing literature on both sides, supporters of “concept learning in animals” seem no closer to persuading the skeptics, while skeptics are no closer to persuading proponents. This rift hinges on disagreements over the strength of the evidence.

Results from dichotomous classification procedures represent the weakest possible evidence for concepts in animals, for reasons unrelated to the validity of corresponding theoretical claims. One pitfall is the *tailor-made classifier*, which may arise during training. Effectively, “teaching to the test” undermines claims about animals' general knowledge. Another is the *lucky guess* during testing. A simplistic response during the testing phase will yield many rewards due to guessing alone, making it difficult to assess the precise content of learning. These shortcomings are independent, such that either might confound an experiment.

### 1.1. The tailor-made classifier

The risk of animal subjects “outsmarting” their minders has been with us since Clever Hans. Whatever the aims of our experimental paradigms, the influence of extraneous information must be minimized so that results reflect the intended empirical test.

Concept learning presents the scrupulous researcher with a challenge: How does one identify (much less control for) the extraneous features of a stimulus? Our understanding of how the brain categorizes stimuli remains limited (Freedman and Assad, 2011), but there is also no consensus about what constitutes a *feature*. The list of stimulus attributes that might be used to categorize stimuli includes overall descriptive statistics (“presence of the color green”), low-level structural details (“T-shaped edge junctions”), patterning (“presence/absence of tiled features”), functional interpretation (“looks like food”), ecological indicators (“bright color = poison”), and interacting levels of analysis (cf. Spalding and Ross, 2000; Marsh and MacDonald, 2008). As such, the content of learning is subject to multiple interpretations.

A *classifier* is an algorithm (however simple or complex) that matches a stimulus with a discrete category. In general, classifiers must undergo training to become sensitive to category-relevant features. Any classifier is limited in what it can detect, and some begin with innate knowledge (such as instincts that some stimuli are threatening). These characteristics hold both for computer algorithms and for the processes used by organisms to classify stimuli. The aim of studying how organisms solve problems of this kind is to discover and describe their classifiers, and to distinguish processes that have evolved recently from those that are well-preserved across many species.

Herein lies the problem: When training requires that only two categories be identified, then the classifier (and therefore the organism) need *only* identify some difference that distinguishes them, and nothing more. The result is a tailor-made classifier: Tailored by the specifics of the binary training paradigm, and optimized solely for that dichotomous discrimination. Just as a bespoke suit is tailored upon request to fit a single person, a tailor-made classifier is only effective at the discrimination it was trained for. At its most extreme, this is Clever Hans in a nutshell: A (cognitively) cheap trick that yields rewards but falls short of generalized knowledge.

When faced with this problem, researchers often narrow the scope of the features available. A set of images might have colors removed, luminances matched, occluders introduced, and noise added (e.g., Basile and Hampton, 2013). Such studies are valuable because they help reveal which features can be used by the classifier. However, regularized stimuli cannot rule a tailor-made classifier, because so many potential “features” might provide the basis for the classification. Furthermore, insofar as the resulting stimuli are “unnatural,” they generalize poorly to how stimuli are categorized in ecological contexts. So long as any feature is consistent enough across stimuli to permit classification, there is a possibility that the classifier relies exclusively on that feature. To be sure that the classifier used by an organism is capable of generalized knowledge, it is essential that training encompass more than a single dichotomous categorization.

### 1.2. The lucky guess

Independent of the classifier (tailor-made or otherwise), the clarity of the evidence depends on how learning is eventually tested. If subjects make dichotomous choices (e.g., “face” vs. “house,” or “same” vs. “different”), then a naive animal will be rewarded half the time. If the positions of the stimuli are counterbalanced (and they usually are, to prevent bias), then this naive animal needn't even randomize its responses; uniformly and insensitively choosing “left” yields a steady stream of rewards.

If a subject's classifier functions even modestly, this rate of reward can be exceeded. However, it is difficult to assess what proportion of correct responses are genuine classifications and what proportion are merely *lucky guesses*. Accuracy of 70% on a binary test could mean that the subject is guessing at random more than half the time (e.g., 40% correct classifications, 30% lucky guesses, 30% unlucky guesses). If a 50% reward rate is deemed satisfactory to the subject, then responding quickly and mindlessly may prove the most favorable strategy. High guessing rates undermine the researcher's ability to make general statements about stimulus properties, particularly given the difficulty in determining which characteristics are used by the classifier.

Guessing is much less effective when tests require more complex responses. If a subject must take a set of *n* stimuli and assign each to one of *n* categories, the odds of guessing correctly drop as *n* increases. This has two benefits. First, error rates will provide an improved signal-to-noise ratio in trying to evaluate the characteristics of a subject's classifier, effectively making every correct sequence of responses more informative. Second, the reward gradient will be better correlated with accuracy: Poor performance will yield far fewer rewards, providing an incentive to attend to the task and to produce high-quality responses.

## 2. A demonstration by simulation

These two confounding factors are relevant regardless of the complexity of the classifier. In education (as in machine learning), teaching to the test yields poor general learning and T/F exams do not reliably measure depth of learning. We offer a concrete simulation using the *bag-of-features* classifier (O'Hara and Draper, 2011) provided in the Computer Vision System toolbox for Matlab v2014b (MathWorks, 2014). Despite relying on low-level features, this approach performs well with photographic stimuli. To represent a “cognitive limit,” we limited all classifiers to no more than 100 clusters of features. The Caltech-101 image bank provided 9665 stimuli belonging to 102 categories (Fei-Fei et al., 2007). Half the images were used to train the classifier, and the other half were set aside as a novel “validation set” for testing.

### 2.1. Training a tailor-made classifier

**Figure 1:**
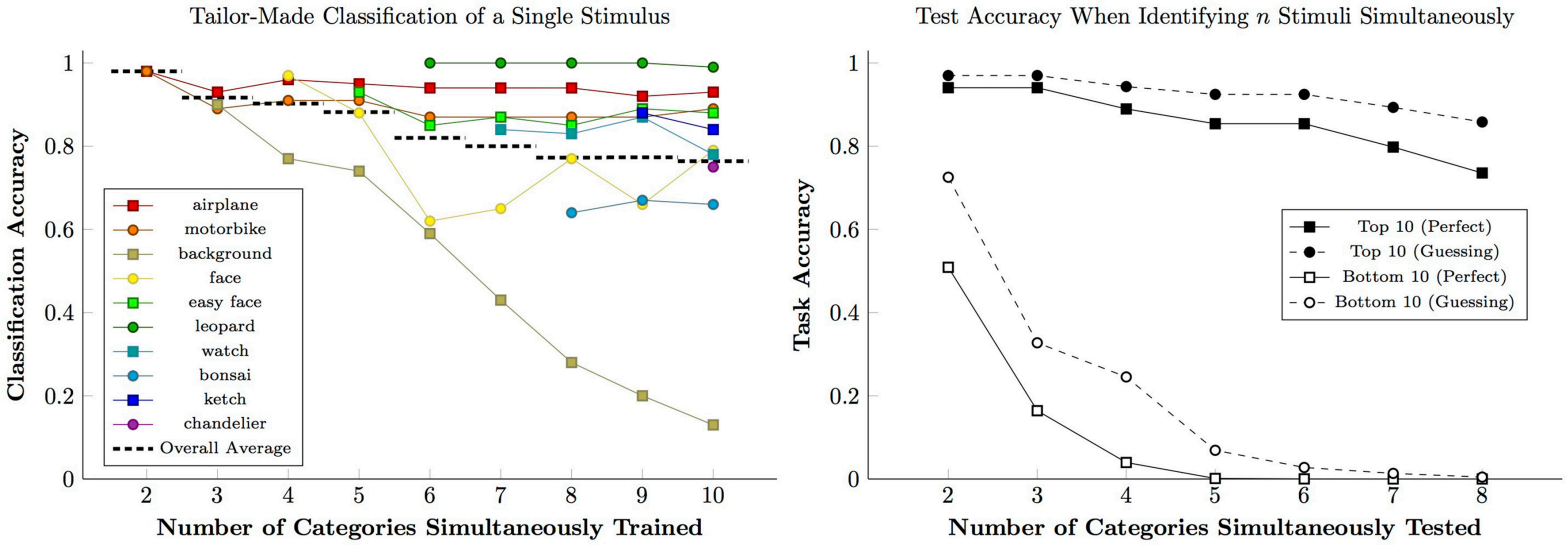
**Performance of the bag-of-features classifier using 100 feature clusters**. **(Left)** Classification accuracy given training on the 10 largest categories in the Caltech 101 sample set. Colored lines show accuracy for specific categories, while dashed black lines show overall accuracy for each level of training complexity. **(Right)** Accuracy by a classifier trained on 102 categories during a test in which *n* stimuli must be classified correctly for a trial to be “correct.” Performance for the classifier's 10 best (black) and worst (white) categories was gauged. Solid lines indicate cases in which classification was done perfectly, while dashed lines indicate cases where correct responses required at least one guess.

The 10 categories in the Caltech 101 with the most images were ordered by size. The classifier was trained and subsequently validated using the first two of these categories, then the first three, and so forth up to ten. Because the classifier was limited to 100 clusters, its criteria became more general as the number of categories increased. The accuracy for each category, as well as the overall average, is plotted in Figure 1 (left).

Some image categories continue to perform well as additional categories are added: Airplanes, leopards, and “easy” faces were categorized correctly over 85% of the time. However, other categories did less well when the classifier was forced to generalize. In particular, performance for the category of “background images” steadily deteriorated, presumably due to the lack of consistent discrete features. If this algorithm was being studied with only three categories, however, this deficiency would not be apparent: Backgrounds were categorized correctly 90% of the time when competing with only two other possible choices, in which case the algorithm identifies them by process of elimination. It is only when backgrounds compete with many other stimuli that the algorithm reveals its inability to handle abstraction.

### 2.2. Measuring the benefits of guessing

We retrained the classifier using all 102 categories. The classifier was then tested as follows: For each trial, exactly one novel image was drawn from each of *n* categories. The classifier had to match *every* image with its corresponding category. Responding randomly succeeds with a frequency of $\frac{1}{n!}$. If classifiers judged multiple images to belong to the same category, one stimulus was randomly assigned to the identified category, while the rest were assigned to whichever categories remained unaccounted for. For example, if pictures of a bonsai and a forest were both identified as “bonsai” rather than as “bonsai” and “background,” respectively, one of the two pictures would be assigned to “bonsai” and the other, by process of elimination, would be assigned to “background.”

Figure 1 (right) shows performance for the classifier's 10 highest-performing categories (black) and ten lowest-performing categories (white). Solid lines show the trials in which every stimulus was correctly identified without guessing, while dashed lines show those trials that were correct given at least one guess. Although there is a clear distinction between high and low performance, there is always a benefit for guessing. In the two-item test, a poor classifier guessed its way to 72% accuracy, a level that would be considered “high” in many published studies. In four- and five-category tests, most rewarded trials for the poor classifiers involved some guessing.

There is a temptation to identify correct responses as indicative of mastery, but even a poor classifier providing vague hunches permits performance to exceed chance. This ‘slightly-informed guessing’ is responsible for many correct responses made by a poor classifier. The best defense against guessing is to increase the complexity of the test, which makes each trial much more informative.

## 3. Recommendations

Our simulation demonstrates perils of narrow category training and of simplistic tests. When a subject (or an algorithm) is trained on only a handful of categories, learning may overspecialize, failing to capture the classifier's general aptitudes. Similarly, when even a highly general classifier is tested on only a few categories at a time, correct trials frequently result from informed guessing rather than from robust representation of all extent categories. We demonstrate these problems separately, but the two can easily act in concert. When a study displays both confounds, it is nearly impossible to judge whether performance arises from any abstract understanding of the stimuli, even when sophisticated *post-hoc* analysis is employed.

The best defense against the possibility of a tailor-made classifier is to increase the number of categories that are trained in parallel. Demonstrating that two-category training transfers to novel stimuli from those categories provides no protection against this problem, since it is fundamentally a problem of how training unfolded. However, while clever tricks may permit pictures of faces to be distinguished from pictures of houses, such trickery is more difficult given three categories, still more difficult given four, and so on.

Many studies that trained more than two categories (e.g., Herrnstein et al., 1976; Sigala, 2009; Vonk, 2013) nevertheless tested only one or two stimuli at a time. Others have required that subjects match a stimulus to one of four categories (Bhatt et al., 1988; Lazareva et al., 2004). Although an improvement, such match-to-sample procedures reward random responses on $\frac{1}{n}$ trials, and informed guessing remains an effective approach for a poor classifier.

Contrary to the recommendations of Katz et al. (2007), we recommend that test conditions require subjects to identify more than one stimulus category during each trial. Unfortunately, few validated methods provide an appropriate level of response complexity. One candidate is the simultaneous chain (Terrace, 2005), which has been used to test serial and numerical cognition. In such cases, subjects would need to choose *n* simultaneously-presented items in a particular order, which requires that subjects process and select from all categories at once. Another candidate is the ALVIN procedure (Washburn and Gulledge, 1995). This task, which requires subjects to imitate a sequence in a manner similar to ‘Simon Says,’ could be adapted to test a classifier by presenting a sequence using one set of stimuli and requiring the imitated sequence to be made using different (but categorically consistent) stimuli. Recovering from the weaknesses of prior concept studies will require that researchers raise the bar, and give their animal subjects the opportunity to succeed (or fail) on their own cognitive merits.

## Author contributions

GJ and DA both contributed the conceptualization, analysis, and writing of this piece.

### Conflict of interest statement

The authors declare that the research was conducted in the absence of any commercial or financial relationships that could be construed as a potential conflict of interest.
